# Trends in Tobacco Smoking in Pregnant Women: Data From French National Perinatal Surveys

**DOI:** 10.3389/ijph.2021.602873

**Published:** 2021-04-01

**Authors:** Virginie Demiguel, Béatrice Blondel, Camille Bonnet, Viêt Nguyen-Thanh, Marie-Josèphe Saurel-Cubizolles, Nolwenn Regnault

**Affiliations:** ^1^ Santé Publique France, French National Public Health Agency, Saint-Maurice, France; ^2^ Université de Paris, CRESS, INSERM, INRA, Paris, France; ^3^ INSERM U1153, Obstetrical, Perinatal and Paediatric Epidemiology Research Team (Epopé), Paris, France

**Keywords:** smoking cessation, surveillance, trends, pregnancy, tobacco smoking

## Abstract

**Objectives:** To describe maternal smoking trends in France between 1972 and 2016, and identify whether maternal characteristics associated with smoking in the 3rd trimester of pregnancy evolved between 2010 and 2016.

**Methods:** Using French National Perinatal Surveys, we estimated proportions of smokers and the number of cigarettes smoked both just before pregnancy and during the 3rd trimester from 1972 to 2016. We used a Poisson model with robust variance to estimate prevalence ratios for smoking during pregnancy.

**Results:** Proportions of mothers quitting smoking were relatively stable (46.0% in 1972 and 45.8% in 2016). The number of cigarettes smoked just before pregnancy and in the 3rd trimester decreased from 1995 onward. However, proportions of smokers remained high before (30.1%) and during the 3rd trimester in 2016 (16.2%). Smoking in the 3rd trimester was associated with a lower education level and lower income in both 2010 and 2016, whereas the association with age, country of birth and parity varied according to the survey year.

**Conclusion:** Early targeted interventions are needed for smokers who plan to have a child and must take smokers’ characteristics during pregnancy into account.

## Introduction

Tobacco smoking in women is a serious public health problem in Europe, with a prevalence well above the average in developed countries [1]. France has one of the highest prevalences of smoking in Europe both for women of childbearing age [2] and pregnant women [3]. Smoking prevalence in France remained stable between 2010 and 2016 in women aged 25–44 (with just over one-third smoking tobacco on a daily basis) [4, 5].

In the most recently published national data available on smoking during pregnancy in France, 20.8% of women declared smoking during the 3rd trimester in 2003. Only 41.8% reported quitting smoking during pregnancy [6], despite the French National Authority for Health’s (*Haute Autorité de Santé*) recommendation not to smoke during this period (www.has-sante.fr). Reported proportion of women quitting smoking during pregnancy differ according to region, and range from 25% in Australia to over 50% in the United States [7, 8].

The harmful effects of smoking during pregnancy on the health of both the mother and the child are well documented. For the former, the risk of developing any one of a large number of associated diseases and pregnancy-related complications (ectopic pregnancy, miscarriage, placenta praevia, etc.) is increased [9]. For the latter, intrauterine growth retardation, preterm delivery and fetal death have all been documented, as well as increased risk of unexpected sudden death [9, 10]. In children and young adults, reduced educational and cognitive abilities [9, 11] and behavioral problems [12] have also been suggested as maternal smoking-related consequences.

Most of the risk factors associated with smoking during pregnancy are well known. These include younger age, a low socioeconomic status, already having children, not living with a partner, and unemployment status [13, 14]. Previous studies have also reported different smoking trends during pregnancy according to sociodemographic groups [15, 16]. This suggests the need for continuous health surveillance of women at greatest risk of prenatal smoking in order to better inform public health policy makers and adjust current smoking cessation interventions. Using data from all six French National Perinatal Surveys (NPS) conducted to date, we aimed to describe temporal trends in smoking prevalence, in the proportions of mothers quitting smoking, and in the number of cigarettes smoked by women just before and during pregnancy, between 1972 and 2016. Our secondary objective was to describe the evolution of the characteristics of women who reported smoking in the 3rd trimester of pregnancy over 2010–2016, as we suppose that the most recent data available are the most relevant to inform future prevention and intervention plans.

## Methods

### Study Design

The NPS are designed to collect information about the health status of mothers and their newborns, perinatal care, maternal behaviour, and risk factors from a representative sample of births. To date six have been conducted: in 1972, 1981, 1995, 2003, 2010, and 2016. All methodological details and study population characteristics have been described elsewhere [5, 17–19].

Briefly, the first two surveys (1972 and 1981) used the same protocol: mainland France was split into 12 zones, the survey taking place each month in a different zone with a sampling fraction twice as small in 1981 as that in 1972. From 1995 onward, the sampling plan was simplified, with the survey being conducted over one week in all public and private maternity units in France. Maternity coverage in all surveys was close to 100% [5].

### NPS Data Collection

Data collection covered all live births and stillbirths with a gestational age of at least 22 weeks or a birth weight of at least 500 g in public and private maternity units. An information letter, describing the objectives and implementation modalities of the survey, was given to women following childbirth by the midwife survey investigator. For those who agreed to participate, data were collected both in a face-to-face interview with another investigator before their discharge, and/or from their medical record. The participation rate was approximately 90% and for most women, data came from both these sources. The interview collected data on sociodemographic characteristics, behaviours just before and during pregnancy-especially maternal smoking–as well as prenatal care. The medical data included information on maternal medical history, delivery and potential adverse outcomes for the newborn.

### Study Sample Size

For the present analyses, the same criteria were used to select the women to include from each NPS (1972–2016) to ensure comparability of indicators over time. Specifically, we included adult women (≥18 years) who gave birth to a live child in mainland France. Furthermore, women who did not answer questions about tobacco use in the NPS (ranging between a minimum of 3.3% in 1972 and a maximum of 9.6% in 2016) were not included in our analyses. Most of these women did not have a face-to-face interview, either because they or their newborn child were in poor health, or because of language difficulties. Accordingly, analyses were based on a sample of 10,474 women in 1972, 5,173 in 1981, 12,219 in 1995, 13,060 in 2003, 13,933 in 2010, and 11,733 in 2016. The size of these samples was sufficient to precisely estimate indicators with a prevalence of at least 10% and to analyse factors related to these indicators. We were granted access to aggregated data for 1972–2003 and individual data for 2010 and 2016 (Supplementary Table S1).

### Variables Studied

#### Variables Relating to Mothers’ Tobacco Consumption

In each NPS, participants retrospectively reported the average number of cigarettes they smoked per day just before pregnancy and during the 3rd trimester. Five categories were created, based on the size of commercial packs of cigarettes: no cigarette, 1–4 cigarettes per day, 5–9 cigarettes, 10–19 cigarettes (or ½ a pack or more), ≥20 cigarettes (or 1 or more packs).

For the most recent years—2010 and 2016—we identified three patterns by comparing the numbers of cigarettes smoked per day just before pregnancy with the number smoked during the 3rd trimester: 1/women who quit smoking during pregnancy (i.e., smoking just before pregnancy but not in the 3rd trimester), 2/women who reduced their consumption during pregnancy, and 3/women who did not reduce or have increased their consumption during pregnancy.

#### Sociodemographic Variables

The following characteristics were studied for both 2010 and 2016: maternal age, country of birth (France, outside France), marital status (living with a partner, single), parity at the time of pregnancy (nulliparous, 1, 2, or ≥ 3), educational level (none/primary/middle school, high school, 1–2 years of tertiary education, 3–4 years or ≥ 5 years of tertiary education), employment status at end of pregnancy (employed, unemployed, housewife, other), average monthly household income (in euros) and type of social health insurance cover at the beginning of pregnancy (general social security cover, other cover (e.g., assistance for undocumented migrants).

### Statistical Analysis

For the first objective, to account for the time trends in tobacco consumption in pregnant women between 1972 and 2016, three indicators were estimated: smoking prevalence just before pregnancy, prevalence during the 3rd trimester, and, the distribution of smokers according to the number of cigarettes smoked per day just before pregnancy and during the 3rd trimester. For these indicators, comparisons between each two consecutive survey year were tested using Pearson’s Chi-square test (with a p-value <0.01 to account for multiple comparisons). For recent years only (2010 and 2016), we also estimated the proportions of smokers who quit, who reduced or who did not reduce/have increased their consumption during pregnancy as a function of their consumption before pregnancy.

For the secondary objective, a Poisson model with robust variance [20] was used to estimate the crude and adjusted prevalence ratios (PR) of smoking in the 3rd trimester of pregnancy and their 95% confidence intervals. This model allowed us to test for the effect of the year on the prevalence of smoking during the 3rd trimester, adjusted for relevant maternal characteristics. All included covariates were categorical variables with the exception of age. For the latter, we used the fractional polynomial method to model the link function of the relationship between maternal age and the prevalence of smoking in the 3rd trimester. Accordingly, maternal age was included as a linear function (as the likelihood ratio test was not significant). To test whether the association of each covariate with the prevalence of smoking in the 3rd trimester changed between 2010 and 2016, an interaction term between the covariate and the study year was introduced into the model. Given the significant interactions observed, we decided to present the results separately for 2010 and 2016.

The analyses were performed using Stata 13.1 software (College Station, Texas 77845 United States).

## Results

Participants in the 2016 NPS were on average 30.3 years old. Most were born in France (81.4%) and lived with a partner (91.6%). Over half were multiparous at the time of pregnancy (57.6%), and had an education level > high school (55.5%). At the end of their pregnancy, two-thirds reported being employed (68.2%) (Supplementary Table S1).

### Tobacco Consumption Prevalence Before and During Pregnancy

**FIGURE 1:**
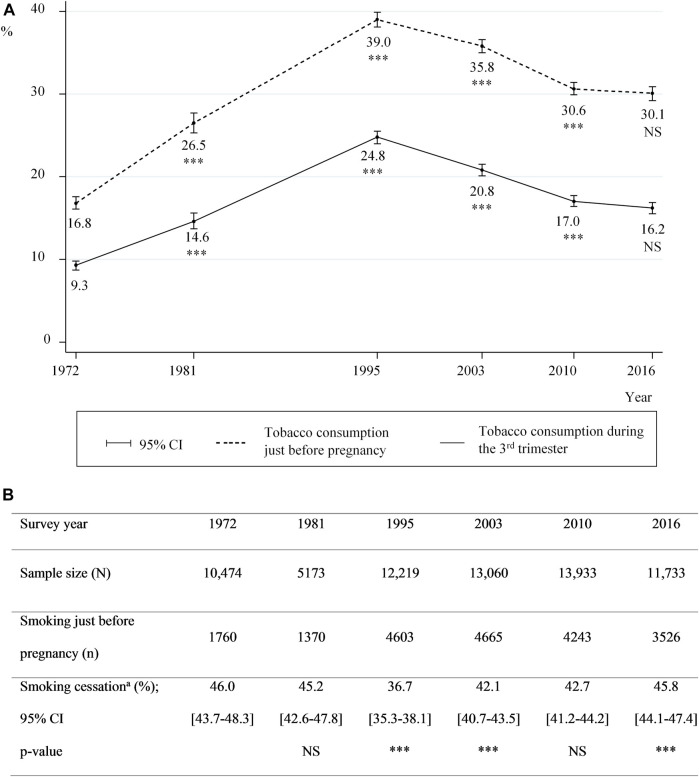
Smoking prevalence before and during pregnancy, and proportions of cessation between 1972 and 2016 **(A)** Smoking prevalence just before pregnancy and during the 3rd trimester **(B)** proportions of smoking cessation; prevalences for two consecutive surveys were compared using Pearson’s Chi-square test **(A)** and **(B)**: 95% CI: 95% confidence intervals; *** for *p*-value<0.01 and NS: not significant **(B)** ^a^Among smokers just before pregnancy (data from the National Perinatal Surveys from 1972 to 2016, France).

In 2016, 30.1 and 16.2% of the NPS participants declared smoking just before pregnancy and in the 3rd trimester, respectively. Although a steady decline in tobacco consumption prevalence during pregnancy was observed from 1995 onward, the difference between 2010 and 2016 was not significant (*p* = 0.08) (Figure 1), even when taking maternal characteristics into account (data not shown). Among women who smoked just before pregnancy, the proportion of those who quit in the 3rd trimester varied little throughout the entire study period (i.e., 1972–2016) (Figure 1).

### Number of Cigarettes Smoked Before and During the 3rd Trimester

**FIGURE 2:**
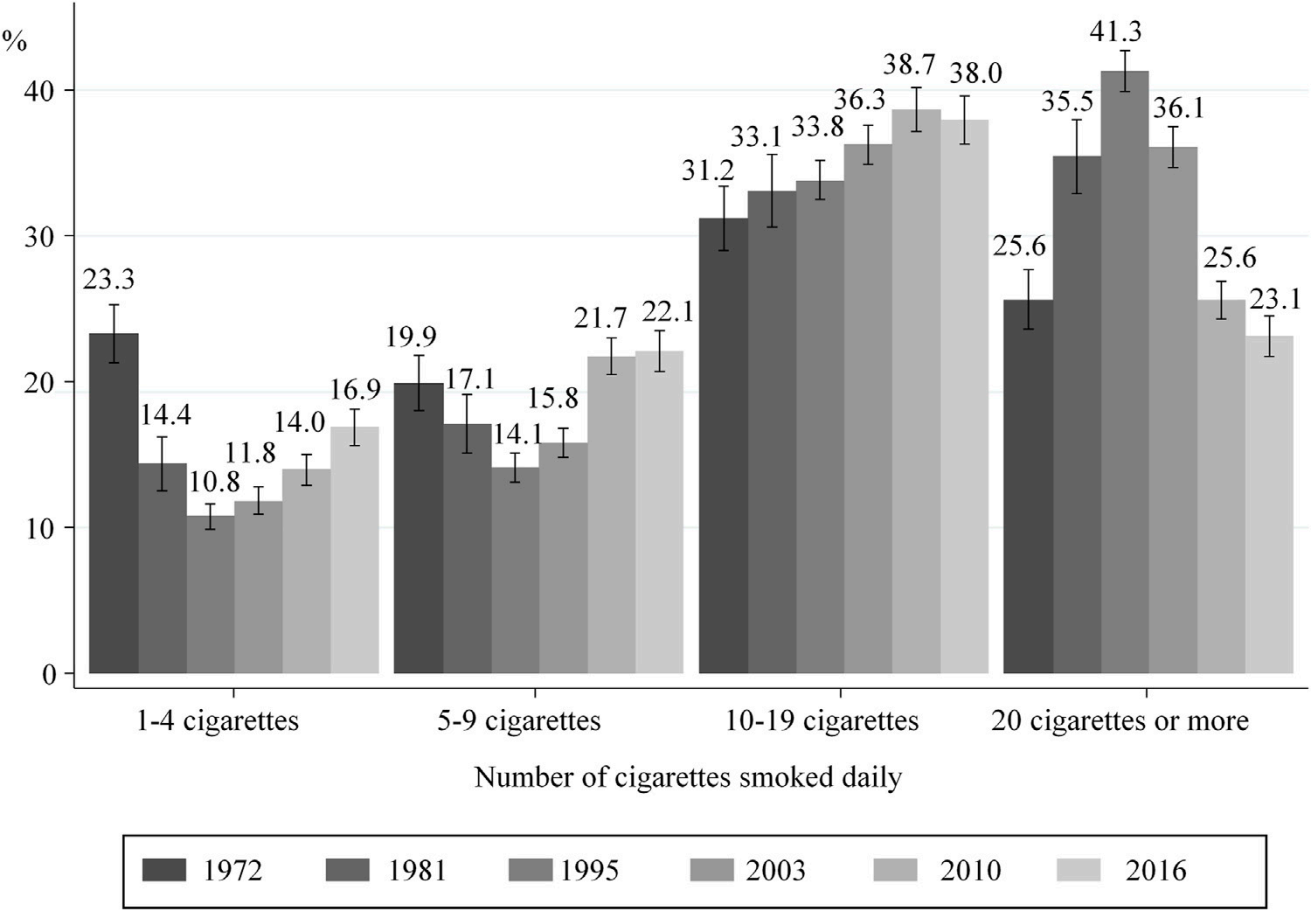
Distribution of smokers according to the number of cigarettes smoked daily just before pregnancy; with 95% confidence intervals (data from National Perinatal Surveys from 1972 to 2016, France).

**FIGURE 3:**
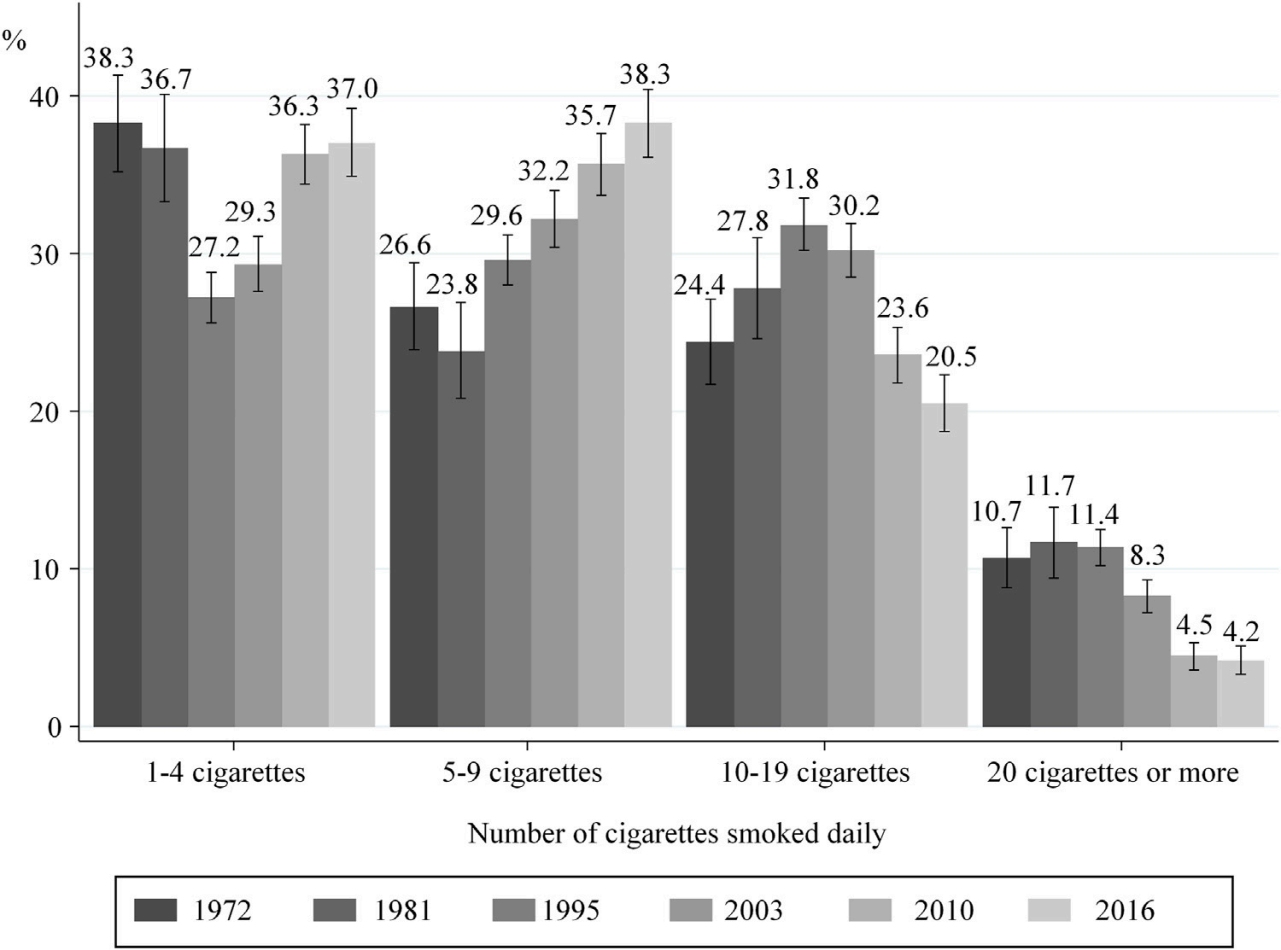
Distribution of smokers according to the number of cigarettes smoked daily in the 3rd trimester of pregnancy; with 95% confidence intervals (data from the National Perinatal Surveys from 1972 to 2016, France).

After an increase in the number of cigarettes smoked daily between 1972 and 1995, lower consumption before pregnancy (Figure 2) and during the 3rd trimester (Figure 3) was observed from 1995 onward. More specifically, the proportion of women smoking an average of ≥20 cigarettes per day in the 3rd trimester decreased from 11.4% in 1995 to 4.2% in 2016, while those smoking an average of 5–9 cigarettes a day increased from 29.6 to 38.3%. However, these proportions remained stable in the last two surveys 2010 and 2016 (Figure 3).

### Evolution of Tobacco Consumption During Pregnancy as a Function of Consumption just Before Pregnancy

**FIGURE 4:**
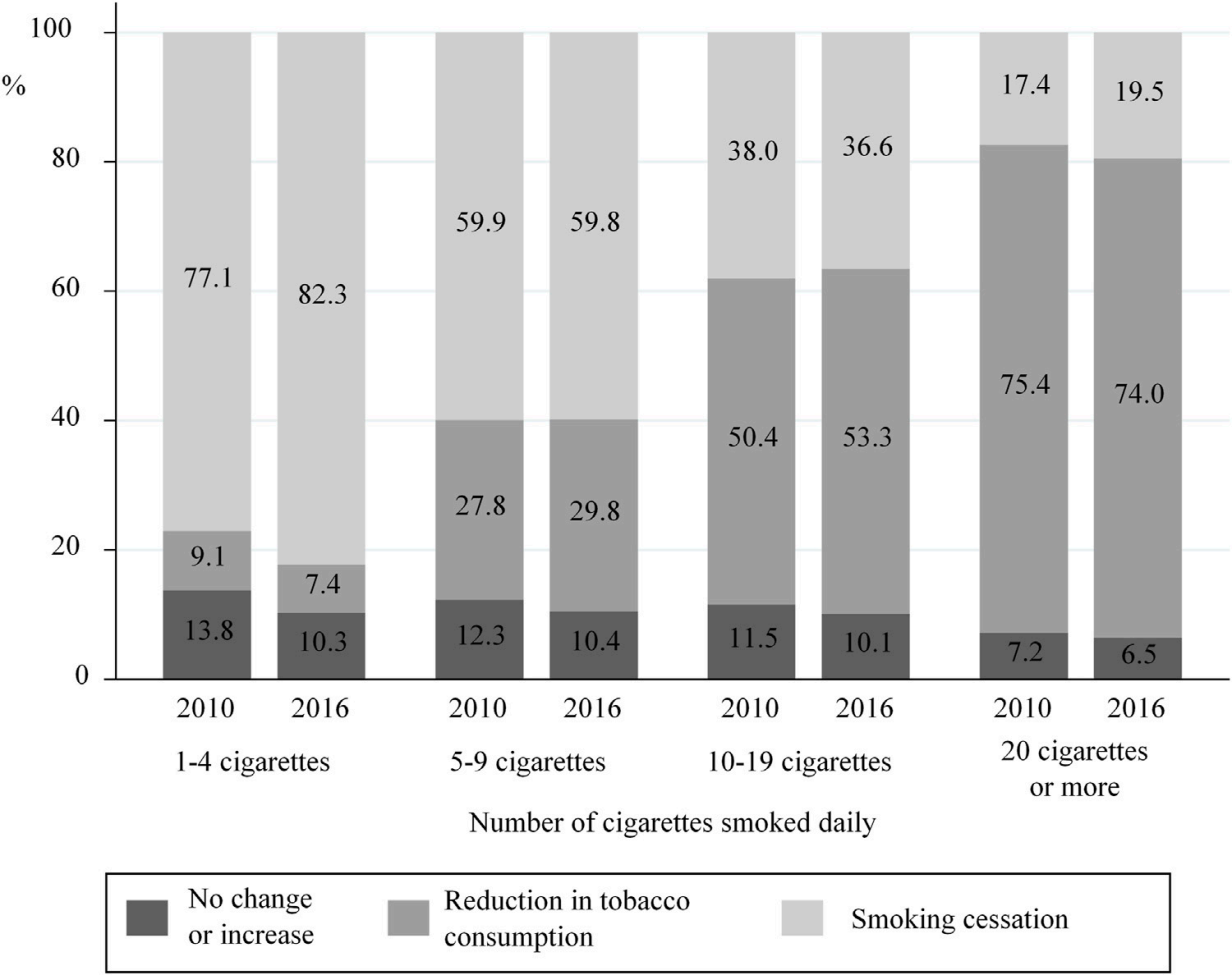
Change between 2010 and 2016 in smoking behaviours (Cessation, reduction, no change) during pregnancy with respect to tobacco consumption smoked just before pregnancy (data from the National Perinatal Surveys in 2010 and 2016, France).

In 2016, 45.8% of women who smoked before pregnancy quit during pregnancy. Nevertheless, the proportion of women quitting smoking varied with the amount of tobacco consumed before pregnancy (*p*< 0.001). More specifically, women who smoked 1–4 cigarettes a day before pregnancy in 2016 were much more likely to quit during pregnancy than those who smoked ≥20 cigarettes/day (82.3 vs. 19.5%) (Figure 4).

In 2016, 44.9% of women who smoked during pregnancy reduced their tobacco consumption compared with their pre-pregnancy consumption (vs. 46.3% in 2010, *p* = 0.21). Of these, 84.8 and 83.1% reduced their consumption by at least 50% in 2010 and 2016, respectively (data not shown). Nevertheless, for both years, the proportion of women reducing their consumption was significantly associated with pre-pregnancy tobacco consumption levels. More specifically, women who previously smoked ≥20 cigarettes a day were ten times more likely to reduce consumption than those who smoked 1–4 cigarettes a day (74.0 vs. 7.4%, respectively). A similar pattern was observed for 2010 (Figure 4). In 2016, only 9.3% of smokers did not decrease their consumption during pregnancy (10.8% in 2010, data not shown).

### Evolution of Characteristics Associated with Tobacco Consumption in the 3rd Trimester of Pregnancy

The proportions of smokers are presented according to mothers’ sociodemographic characteristics for each survey year (Table 1).

**TABLE 1:** Maternal characteristics associated with smoking in the 3rd trimester of pregnancy in 2010 and 2016 (univariate analysis). Data from the National Perinatal Surveys in 2010 (N = 13,777) and 2016 (N = 11,704), France.

National perinatal survey	2010			2016		
	N	Smoking prevalence (%)	*p* value^a^	N	Smoking prevalence (%)	*p* value^a^
Total	13,777	17.0		11,704	16.2	
Maternal age	13,763		<0.001	11,704		<0.001
<25 years old	2,260	26.2		1,501	26.5	
25–29	4,604	17.0		3,719	15.7	
30–34	4,266	13.9		4,008	14.8	
35 +	2,633	14.3		2,476	13.0	
Country of birth	13,689		<0.001	11,703		<0.001
France	11,209	19.7		9,524	18.5	
Outside France	2,480	5.0		2,179	6.1	
Marital status	13,671		<0.001	11,680		<0.001
Living with a partner	12,718	15.9		10,701	15.0	
Single^b^	953	32.2		979	29.4	
Parity at the time of pregnancy	13,709		<0.001	11,700		<0.001
Nulliparous	5,928	16.3		4,963	15.6	
1	4,768	15.3		4,208	15.5	
2	1,967	18.5		1,665	17.0	
3 or more	1,046	26.4		864	21.8	
Educational level	13,642		<0.001	11,604		<0.001
None/Primary/Middle school	3,780	30.1		2,655	32.1	
High school	2704	20.5		2,514	19.6	
1–2 years of tertiary education	2,939	13.1		2,238	12.7	
3–4 years of tertiary education	2,430	7.1		2,110	7.8	
≥5 years of tertiary education	1,789	4.4		2,087	4.2	
Employment status at the end of pregnancy	13,190		<0.001	11,445		<0.001
Employed	9,290	14.1		7,797	13.0	
Unemployed	1,687	27.9		1,921	24.6	
Housewife	1,807	22.9		1,384	21.2	
Other (including student)	406	14.5		343	14.6	
Average monthly household income (€)	13,286		<0.001	11,510		<0.001
<1,000	1,291	28.0		1,101	26.3	
[1,000–1,500]	1,357	24.2		986	24.7	
[1,500–2000]	1,964	22.6		1,457	21.7	
[2000–3000]	4,052	18.0		3,189	18.3	
[3000–4000]	2,782	11.0		2,695	11.3	
4,000 or more	1,840	6.7		2,082	6.5	
Type of social insurance cover at the beginning of pregnancy	13,625		<0.001	11,690		<0.001
General social security	11,765	15.5		10,028	14.8	
Other (AME/CMU/None)^c^	1,860	26.9		1,662	25.1	

The number of missing data varied for each characteristic.

^a^ Pearson Chi-square test.

^b^ single women and women not living with their partner (for 2010, it was not possible to identify women who declared being in a couple but were not living with their partner).

^c^ AME: state Medical assistance for undocumented migrants; CMU: health insurance for people with low or no income.

In 2010 and 2016, the prevalence of smoking in the 3rd trimester was higher in women who lived alone and those not living with their partner. Furthermore, the lower the education and income levels, the higher the prevalence of smoking in the 3rd trimester. These associations did not change between 2010 and 2016 (non-significant interaction tests) (Table 2).

**TABLE 2:** Maternal characteristics associated with smoking in the 3rd trimester of pregnancy in 2010 and 2016 (multivariable analysis), data from the National Perinatal Surveys in 2010 (N = 12,672) and 2016 (N = 11,132), France.

	2010			2016		
	Prevalence ratio	95% CI	p value^a^	Prevalence ratio	95% CI	p value^a^
Maternal age (years)^b^ ^c^	0.97	[0.93–1.01]	0.115	1.06	[1.01–1.11]	0.01
Country of birth^c^			<0.001			<0.001
Outside France	1	-		1	-	
France	4.57	[3.79–5.52]		3.47	[2.89–4.17]	
Marital status			<0.001			<0.001
Living with their partner	1	-		1	-	
Single^d^	1.34	[1.19–1.51]		1.26	[1.11–1.43]	
Parity at the time of pregnancy^c^			<0.001			0.700
Nulliparous	1	-		1	-	
1	1.01	[0.93–1.11]		0.97	[0.88–1.07]	
2	1.09	[0.97–1.23]		0.93	[0.81–1.06]	
3 or more	1.38	[1.20–1.59]		0.94	[0.80–1.11]	
Educational level			<0.001			<0.001
None/primary/middle school	4.82	[3.77–6.16]		5.83	[4.59–7.42]	
High school	3.62	[2.82–4.63]		3.80	[2.90–4.85]	
1–2°years of tertiary education	2.37	[1.85–3.04]		2.50	[1.95–3.20]	
3–4°years of tertiary education	1.42	[1.08–1.85]		1.75	[1.35–2.26]	
≥5°years of tertiary education	1	-		1	-	
Employment status at end of pregnancy			<0.001			<0.001
Employed	1	-		1	-	
Unemployment	1.28	[1.16–1.41]		1.25	[1.13–1.40]	
Housewife	1.06	[0.95–1.19]		1.17	[1.02–1.35]	
Other (including students)	0.78	[0.61–1.00]		0.98	[0.76–1.24]	
Average monthly household income (€)			0.002			0.004
<1,000	1.39	[1.10–1.76]		1.52	[1.28–1.95]	
[1,000–1,500]	1.46	[1.17–1.82]		1.52	[1.21–1.93]	
[1,500–2000]	1.46	[1.18–1.81]		1.44	[1.16–1.79]	
[2000–3000]	1.31	[1.07–1.60]		1.38	[1.14–1.70]	
[3000–4000]	1.14	[0.93–1.40]		1.17	[0.96–1.43]	
4000 or more	1	-		1	-	
Type of social insurance cover at the beginning of pregnancy^c^			<0.001			0.330
General social security	1	-		1	-	
Other (AME/CMU/None)^e^	1.23	[1.10–1.36]		1.02	[0.90–1.16]	

Data were available for 93% of the women in the sample.

^a^ Wald test.

^b^ calculated per 5-years increase in age.

^c^ significant interactions: Survey year*age (p = 0.003); survey year*country of birth (p = 0.04); survey year*Parity (p = 0.004) and survey year*type of social insurance cover (p = 0.03).

^d^ single women and women not living with their partner.

^e^ AME: state medical assistance for undocumented migrants; CMU: health insurance for people with low or no income.

Interaction tests showed that the association between tobacco consumption in the 3rd trimester and maternal characteristics differed according to survey year for age (*p* = 0.003), parity (*p* = 0.004) and country of birth (*p* = 0.04). More specifically, while the prevalence of smoking was not associated with age in 2010, a 6% higher prevalence ratio was observed for each 5 years age increment in 2016. Furthermore, the higher prevalence of smoking in the 3rd trimester in women with three or more children in 2010 (PR = 1.38, 95% CI [1.20–1.59]) was no longer significant in 2016.

The relatively higher prevalence of smoking during pregnancy in women born in France (compared with those born elsewhere) observed in 2010 was also found in 2016, albeit to a lesser degree (PR = 4.57, 95% CI [3.79–5.52] in 2010 versus. 3.47, 95% CI [2.89–4.17] in 2016). More specifically, while the prevalence ratio of smoking during pregnancy in 2016 was lower than in 2010 in women born in France (PR = 0.64, 95% CI [0.47–0.87]), it tended to increase slightly in women born elsewhere (PR = 1.17, 95% CI [0.91–1.50]) (data not shown).

## Discussion

### Principal Findings

Our study shows that between 1995 and 2010, tobacco consumption in women living in France both before and during pregnancy decreased, while proportions tended to stabilize between 2010 and 2016. The proportions of mothers quitting smoking were remarkably constant between 1972 and 2016 with approximately 45% of women quitting smoking during pregnancy. Between 1995 and 2016, the amount of tobacco consumed during pregnancy decreased for those who continued to smoke while pregnant. The prevalence of smoking in the 3rd trimester was higher in women with less favourable socioeconomic characteristics in both 2010 and 2016. In contrast, the association between smoking in the 3rd trimester and age, country of birth and parity differed between both these years.

### Stabilization of smoking prevalence and of the number of cigarettes smoked daily by women living in France before and during pregnancy between 2010 and 2016

Public health measures implemented between 2010 and 2016 in France (increases in the prices of packets of cigarettes, visual health messages on packets, flat-rate annual reimbursement for nicotine replacement treatments (NRT)) had little effect on reducing smoking prevalence and daily tobacco consumption before and during pregnancy over this period. However, complementary measures introduced in 2015–2016—including a pictogram advocating the non-consumption of tobacco during pregnancy on all tobacco products, the total reimbursement of NRT for pregnant women and their close family, plain packaging, ambitious social marketing campaigns and price increases on packets of cigarettes (10 euros by the end of 2020)—may prove to have a greater influence on reducing smoking prevalence in women who are or wish to become pregnant.

### Higher Probability of Quitting Among Light Smokers

Our results show that the probability of quitting during pregnancy was higher when consumption just before pregnancy was low, reflecting findings elsewhere [21]. In addition, heavy smokers tended to reduce the number of cigarettes they smoked daily more than light smokers. This raises questions about the contents of anti-smoking messages and the level of support provided for quitting, especially from healthcare professionals. In the 2016 NPS, 79.9% of women reported that they had been interviewed by a healthcare professional about their tobacco consumption during pregnancy. Furthermore, only 46.3% of those who reported smoking during pregnancy declared receiving counseling for quitting [5]. It is possible that healthcare providers find it difficult to convince and help women to quit. Indeed, French general practitioners are more inclined to advise their pregnant patients to reduce their consumption rather than quit, even for heavy smokers in early pregnancy [22]. This is despite the fact that even moderate consumption is associated with low birth weight [23].

### Interventions in Women of Childbearing Age and Pregnant Women

The proportions of mothers quitting smoking remained stable between 42.1 and 46.0% over the whole study period except in 1995 (when this proportion was 36.7%), while smoking prevalence just before pregnancy varied from 16.8 to 35.8%. This suggests that the prevalence of smoking during pregnancy is directly related to prevalence among women of childbearing age. The literature shows that the number of quit attempts is a positive predictor of a successful subsequent attempt [24] and that the probability of quitting during pregnancy is higher when consumption before pregnancy is low. Accordingly, any attempt to quit, even unsuccessful, in women of childbearing age, may increase the chances of success when they become pregnant [25]. Every general population-based intervention which leads to a reduction in tobacco consumption before pregnancy should also encourage smoking cessation during pregnancy.

Campaigns and interventions specifically targeting pregnant women have also been effective in increasing proportions of mothers quitting smoking during pregnancy [26]. Pregnancy is considered a favourable time for behavioral change, as parents are very receptive to prevention messages [27]. Quitting is beneficial for both the mother and the child, provided the former maintain long-term abstinence [1, 28]. However, according to one literature review, 75% of women relapse at one year after childbirth [29]. Promoting smoking cessation before pregnancy and continued abstinence in postpartum [30] would reduce the development of chronic, and therefore costly diseases both in mothers and their children [31].

### Socioeconomic Characteristics Associated With Smoking During Pregnancy

The prevalence of smoking during pregnancy remained higher in women with less favourable socioeconomic characteristics in 2010 and 2016. This reflects findings in the literature [14, 15]. Health inequalities due to less favourable socioeconomic characteristics might be explained by a lower level of health literacy which leads to less knowledge and understanding of the risks for unborn children, of preventive measures (quitting smoking, taking folic acid, etc.) and of existing support systems [14]. These women may also consider tobacco to be an indispensable tool to manage stress [32]. Furthermore, a higher level of tobacco dependence and the fact that smoking is less frowned upon culturally in France, may also explain the greater difficulty these socially disadvantaged smokers face when attempting to quit [33]. Finally, women with a lower socioeconomic status who wish to stop smoking may need more psychological support and help from healthcare professionals because they receive less social support through advice and encouragement to quit from family and friends [34]. All these issues highlight the need for targeted actions to reduce social inequalities in terms of tobacco consumption [35]. It should be noted that, for these analysis, we hypothesized that marital status, employment status and household income did not change between the 3rd trimester of pregnancy and childbirth as the latter occurs so close to the former.

Unlike 2010, our data for 2016 highlight an increased prevalence of smoking with age in pregnant women. This is consistent with the fact that while the prevalence of daily smoking in women in the general population between both years decreased from 30.0 to 25.2% in 15–24 year olds (*p* < 0.05), it remained stable among 25–34 year olds (33.3% in 2016) and 35–44 year olds (32.5%) [4].

The trend in the prevalence ratio according to place of birth (i.e., France versus outside France) reflects previous findings [15, 16]. In the general French population, country of birth has been associated with tobacco consumption levels [36]. Indeed, non-native born women living in France are less likely to smoke. In our study, we found that the association between smoking during pregnancy and the place of birth varied between 2010 and 2016, with women born outside France gradually adopting French cultural habits (i.e., they tended to start smoking more). Additional analyses on the geographical area of origin and the length of time living in France [37] would make it possible to refine these results.

### Strengths and Limitations

The strengths of our study include repeated surveys over a long observation period (45 years) and its exhaustiveness. With regard to the latter, almost all maternity units and all mothers agreed to participate in the NPS. We compared the number of births with birth certificates recorded by the French National office of vital statistics [38] and these numbers were very similar. Data quality was ensured by using face-to-face interviews with trained midwives. Consequently, few data were missing from the questionnaires. Furthermore, data on the characteristics of mothers, deliveries and newborns were similar to those found in the national statistics provided by hospital discharge summaries [39, 40]. The total sample of each survey is considered representative of annual births. Less than 10% of women did not answer questions about tobacco use. The stability of the design used enables trends comparisons to be made. Finally, the sample size and the broad spectrum of variables collected made it possible to analyse changes in tobacco consumption according to many maternal characteristics in 2010 and 2016.

One limitation of the study is that women who refused or could not participate in the NPS, were excluded from our analyses. These included women who had given birth to a stillborn child and those who were not interviewed because of language barriers. This may have introduced selection bias. However, stillbirths are relatively uncommon in developed countries and therefore would have had a low impact on our estimated smoking prevalences. [3]. Furthermore, language barriers may have contributed to an under-representation of mothers not born in France (18.1% of women in our study sample versus. 22.8% in the French National office of vital statistics [38]. However, with regard to the study of risk factors associated with tobacco use, this limitation may be less relevant since women born outside France are less likely to smoke than their native-born French counterparts [41].

Another limitation in the NPS—and therefore in the present study—is that tobacco consumption is self-reported. No objective measurement is made in the NPS (e.g., using different markers such as saliva cotinine concentration [42, 43]. Higher proportions of non-disclosure of smoking by pregnant women have previously been reported [44]. Furthermore, very light smokers and those who smoke for a very brief time during pregnancy are the women most likely to underreport their tobacco consumption [45]. Nevertheless, previous findings have shown that studies which do not focus specifically on tobacco—like the NPS—described lower underreporting. [46, 47]. The NPS aim to characterize all indicators related to pregnancy in France and not just smoking behaviour. As a result, only a limited number of questions were asked about this specific topic. For instance, we had no information about the smoking status of partners, the exact date of smoking cessation during pregnancy, or the motivations that drove women to quit or reduce consumption.

### Conclusions and Implications for Policy and Practice

In France, tobacco consumption before and during pregnancy remained high over the study period. Although the amount of tobacco consumed decreased from 1995 onward, the proportions of cessation during pregnancy remained stable from 1972 onward. Cessation remains a major public health issue [23]. In order to help reduce the burden of smoking during pregnancy, campaigns and practical support for the general population are needed. These must be complemented by more specific actions targeting pregnant women, in particular women at a greater risk of continuing to smoke during pregnancy. Providing initial and continuous training to healthcare providers may also help integrate smoking cessation—not only reduction—into routine prenatal care. To help evaluate and guide such policies, it is also important to continue monitoring the trends in tobacco smoking in pregnant women and the associated risk factors, especially in France where several ambitious tobacco control measures have been implemented since 2016.

## Data Availability

The data analyzed in this study is subject to the following licenses/restrictions: The data that support the findings of this study are not publicly available. A reasonable request must be submitted to Obstetrical, Perinatal and Pediatric Epidemiology Research Team EPOPé, INSERM U1153 and approved by the French Data Protection Authority (CNIL). Requests to access these datasets should be directed to epope@inserm.fr.
