# Supplementary material for: Trends in Tobacco Smoking in Pregnant Women: Data From French National Perinatal Surveys
Source: Int J Public Health. 2021 Apr 1;66:602873. doi: 10.3389/ijph.2021.602873 (PMC8565271; doi:10.3389/ijph.2021.602873)
Supplement: Supplementary file 1 [file Table1.docx]

**Supplementary File 1** Sociodemographic characteristics of mothers of the study population (data from the National Perinatal Surveys in 2010 (N = 13,933) and 2016 (N = 11,733), France)

| National Perinatal Survey | 2010 | | 2016 | |  |
| --- | --- | --- | --- | --- | --- |
|  | (N=13,933) | | (N=11,733) | |  |
|  | n | (%) | n | (%) | p-value^a^ |
| Maternal age | 13,919 | | 11,733 | | <0.001 |
| <25 years old | 2296 | (16.5 %) | 1504 | (12.8 %) |  |
| 25-29 | 4655 | (33.4 %) | 3723 | (31.7 %) |  |
| 30-34 | 4309 | (31.0 %) | 4020 | (34.3 %) |  |
| 35 + | 2659 | (19.1 %) | 2486 | (21.2 %) |  |
| Maternal age (years) – mean (sd) | 29.7 (0.04) | | 30.3 (0.05) | | <0.001 |
| Country of birth | 13,827 | | 11,732 | | 0.314 |
| France | 11,323 | (81.9 %) | 9551 | (81.4 %) |  |
| Outside France | 2504 | (18.1 %) | 2181 | (18.6 %) |  |
| Marital status | 13,814 | | 11,709 | | <0.001 |
| Living with their partner | 12,851 | (93.0 %) | 10,729 | (91.6 %) |  |
| Single^b^ | 963 | (7.0 %) | 980 | (8.4 %) |  |
| Parity at the time of pregnancy | 13,865 | | 11,729 | | 0.222 |
| Nulliparous | 6010 | (43.3 %) | 4978 | (42.4 %) |  |
| 1 | 4815 | (34.7 %) | 4218 | (36.0 %) |  |
| 2 | 1987 | (14.3 %) | 1667 | (14.2 %) |  |
| 3 or more | 1053 | (7.6 %) | 866 | (7.4 %) |  |
| Educational level | 13,786 | | 11,633 | | <0.001 |
| None/Primary/Middle school | 3826 | (27.8 %) | 2662 | (22.9 %) |  |
| High school | 2741 | (19.9 %) | 2518 | (21.6 %) |  |
| 1-2 years of tertiary education | 2966 | (21.5 %) | 2245 | (19.3 %) |  |
| 3-4 years of tertiary education | 2457 | (17.8 %) | 2118 | (18.2 %) |  |
| ≥5 years of tertiary education | 1796 | (13.0 %) | 2090 | (18.0 %) |  |
| Employment status at end of pregnancy | 13,327 | | 11,471 | | <0.001 |
| Employed | 9384 | (70.4 %) | 7818 | (68.2 %) |  |
| Unemployed | 1708 | (12.8 %) | 1926 | (16.8 %) |  |
| Housewife | 1825 | (13.7 %) | 1384 | (12.1 %) |  |
| Other (including student) | 410 | (3.1 %) | 343 | (3.0 %) |  |
| Average monthly household income (€) | 13,418 | | 11,539 | | <0.001 |
| <1000 | 1310 | (9.8 %) | 1102 | (9.6 %) |  |
| [1000-1500[ | 1368 | (10.2 %) | 988 | (8.6 %) |  |
| [1500-2000[ | 1981 | (14.8 %) | 1462 | (12.7 %) |  |
| [2000-3000[ | 4103 | (30.6 %) | 3198 | (27.7 %) |  |
| [3000-4000[ | 2803 | (20.9 %) | 2700 | (23.4 %) |  |
| 4000 or more | 1853 | (13.8 %) | 2089 | (18.1 %) |  |
| Type of social insurance cover at the beginning of pregnancy | 13,767 | | 11,719 | | 0.259 |
| General Social Security | 11,881 | (86.3 %) | 10,056 | (85.8 %) |  |
| Other (AME/ CMU / None)^c^ | 1886 | (13.7 %) | 1663 | (14.2 %) |  |

The number of missing data varies for each characteristic

a: Pearson Chi-square test

b: single women and women not living with their partner (for 2010, it was not possible to identify women who declared being in a couple but were not living with their partner)

c: AME: state Medical assistance for undocumented migrants; CMU: health insurance for people with low or no income
